# Joint Dysfunction as a Cause of Spontaneous Subclinical Bleeding in Infants with Hemophilia

**DOI:** 10.3390/jcm12206672

**Published:** 2023-10-22

**Authors:** Elena Anna Boccalandro, Samantha Pasca, Valentina Begnozzi, Roberta Gualtierotti, Pier Mannuccio Mannucci

**Affiliations:** 1 Fondazione IRCCS Ca’ Granda Ospedale Maggiore Policlinico, Angelo Bianchi Bonomi Hemophilia and Thrombosis Center, 20122 Milan, Italy; boccalandro.elena@gmail.com (E.A.B.); valentinabegnozzi@yahoo.it (V.B.); piermannuccio.mannucci@policlinico.mi.it (P.M.M.); 2 Biomedical Sciences Department (DSB), Padua University Hospital, 35131 Padua, Italy; sampasca27@gmail.com; 3 Department of Pathophysiology and Transplantation, Università degli Studi di Milano, 20122 Milan, Italy

**Keywords:** hemophilia, joint, gait, synovitis

## Abstract

Hemophilia is an inherited hemorrhagic disorder; its main clinical manifestations being bleeding in muscles and joints. Ankles, knees, and elbows are the most frequently affected joints, followed by shoulders and hips. The clinical signs of joint involvement are reduced mobility, swelling and walking difficulties. Bleeding episodes in patients with hemophilia are usually divided into traumatic and spontaneous, but we believe that the latter are not truly spontaneous but rather the result of joint stresses owing to motion actions that create dysfunctions starting from infancy. Pharmacological prophylaxis with factor replacement therapies or non-replacement drugs markedly reduces musculoskeletal hemorrhages. However, the onset of subclinical joint stress can be reduced only by associating this therapeutic approach with the accurate observation of the child motion patterns and restoring them if dysfunctional, thereby primarily preventing subclinical bleeding and ultimately the onset or progression of hemophilic arthropathy.

## 1. Introduction

Congenital hemophilia is a rare hemorrhagic disorder characterized by the deficiency of coagulation factor VIII (FVIII) in hemophilia A or factor IX (FIX) in hemophilia B. The severity of the disease depends on the plasma levels of FVIII or FIX and is therefore classified as mild (deficient factor 6–40%), moderate (1–5%), or severe (<1%). The standard of care is prophylactic treatment with clotting factor concentrates or non-substitutive drugs such as emicizumab and others currently in the development pipeline, except for milder cases which in general need and adopt episodic therapy only at the time of bleeding or before surgical interventions [1].

Typical clinical manifestations of hemophilia are recurrent bleeds, causing organ damage and requiring for management a multidisciplinary approach. Hemorrhagic events mainly occur in the musculoskeletal system, especially in joints [2,3]. Ankles, knees, and elbows are those most frequently affected, followed less frequently by shoulders and hips. After the occurrence of three or more bleeds at a single joint within a consecutive 6-month period, the joint is referred to as the target joint. The clinical signs of joint involvement are walking difficulties, reduced mobility and swelling due to palpable synovial hypertrophy, but also, in the long-term, muscle and capsular contractures and disabling arthropathy [4,5]. In hemophilia, musculoskeletal bleeding has been classified as traumatic or spontaneous, the latter affecting almost exclusively patients with severe or moderately severe disease [1]. But what is the real cause of these bleeds? Are they truly spontaneous as generally claimed?

## 2. How Joint Stress Causes Dysfunction and Bleeding

We suggest that in infants with severe hemophilia, joint bleeding (hemarthrosis) does not occur spontaneously but rather is the response to repeated stress on the joint at the time of well-defined motion actions, and that the phenomenon commonly known as spontaneous hemarthrosis is the result of repeated mechanical stress. The most clinically evident manifestations occur as muscle or joint bleeding as soon as the infant begins to move and walk. These hemorrhagic events are localized more frequently in elbows, knees, and ankles, the main support joints during the motion activities of the infant. Mastering the sitting position and the curiosity associated with the need to reach nearby objects are the mechanisms that favor early psychomotor development and then, step by step, the early beginning of functional motion strategies. Functional motion strategies are chosen by the infant with hemophilia also in case of subclinical joint micro-bleeds [6]. The accompanying pain caused by continuous mechanical stress may not be noticed by parents or physicians, not only because infants are not able to verbalize it but also because the central nervous system instructs the musculoskeletal system to adopt compensatory strategies such as antalgic gait or even complete refusal to walk in order to avoid pain in the affected limb. In our experience, these alternative strategies related to early motion activities, if inadequately corrected, imply patterns of motion that are less efficient and ergonomic than the correct ones and create the conditions for the onset of dysfunction of the whole musculoskeletal system. A disharmonious motion, that implies an unbalanced solicitation of the two limbs favoring the use of one to the detriment of the other, easily leads to the onset of a functional dysfunction. Therefore, when a child with hemophilia starts walking, the conditions for the first hemarthrosis are already set and further increase the risk of bleeding in that joint—the so-called target joint—and eventually lead to irreversible damage.

During infancy, the elastic component is much more prominent than the fibrotic one in the musculoskeletal system [7]. Thus, in the first few months of living with hemophilia, the appearance of subcutaneous or muscle bleeds involving the connective component of the musculoskeletal system is more frequent at a time when joint bleeds are still absent. Then, at the end of the first year of life, especially when the child begins to acquire the upright position, joint pressure increases, especially in knees and ankles, and the first clinically evident joint bleed starts to appear. Hemarthrosis in the elbows is usually less frequent, except when these joints have been excessively stressed during the crawling phase of infant development. 

Young patients with hemophilia show functional impairment even though asymptomatic. A study by Stephensen et al. [8] reported that young boys (7–13 years old) with hemophilia show alterations in the kinematic parameters compared with normal healthy age-matched controls. In addition, Seuser et al. [9] observed reduced overall fitness, endurance, coordination, and flexibility in children with hemophilia.

With this background and preambles, it can therefore be hypothesized that the target joints are the first that undergo the greatest stress that, owing to pain, leads to the implementation of an alternative functional strategy that allows the joint to be safeguarded, using alternative functional resources that the musculoskeletal system makes available. During infancy and until the complete maturation of the musculoskeletal system, the available elastic resources compensate well for this problem. However, unless the musculoskeletal system is helped to recover these resources, alternative motion strategies are implemented, thereby introducing dysfunctional motion modalities that often cause damage to the whole musculoskeletal system [4,10].

## 3. How to Detect the Problem

Parents are the first adult figures that an infant meets, and thus they are those who first must be able to recognize each small problem of their children. They must be able to interact early by offering them the correct motion activities from the first few months of life so that the infant will be able to fully develop the musculoskeletal functions and progressively reach in due time all the main developmental stages. Experience, consisting of a set of motion activities proposed to the infant, plays a fundamental role in learning the skills necessary to reach adequate metrics of motion development. Experience is not mainly determined by individual features but rather by the methods of care offered during the different stages of child growth: from the usual daily activities to games and stimuli that the caregiver should offer every day to the child. Therefore, if parents are aware of the importance of tummy time and offer to the infant the right motion activities from the first few months of life, the child will develop the best and reach all the main developmental stages. Conversely, if parents make or allow frequent postural errors and offer too few motion activities, the child risks slowing down development and skipping fundamental milestones in the frame of motion development. 

**Figure 1:**
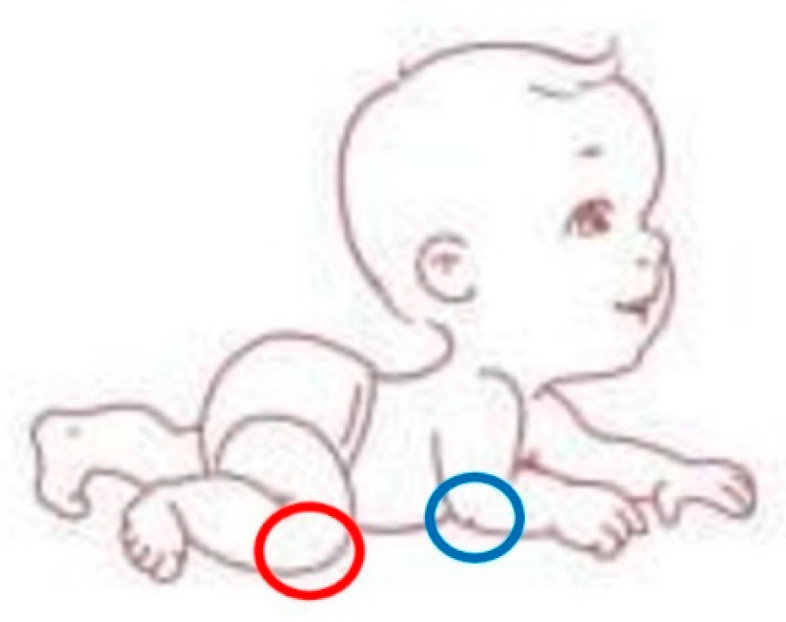
The figure shows how during crawling knees and elbows are the most stressed joints (in red and blue).

**Figure 2:**
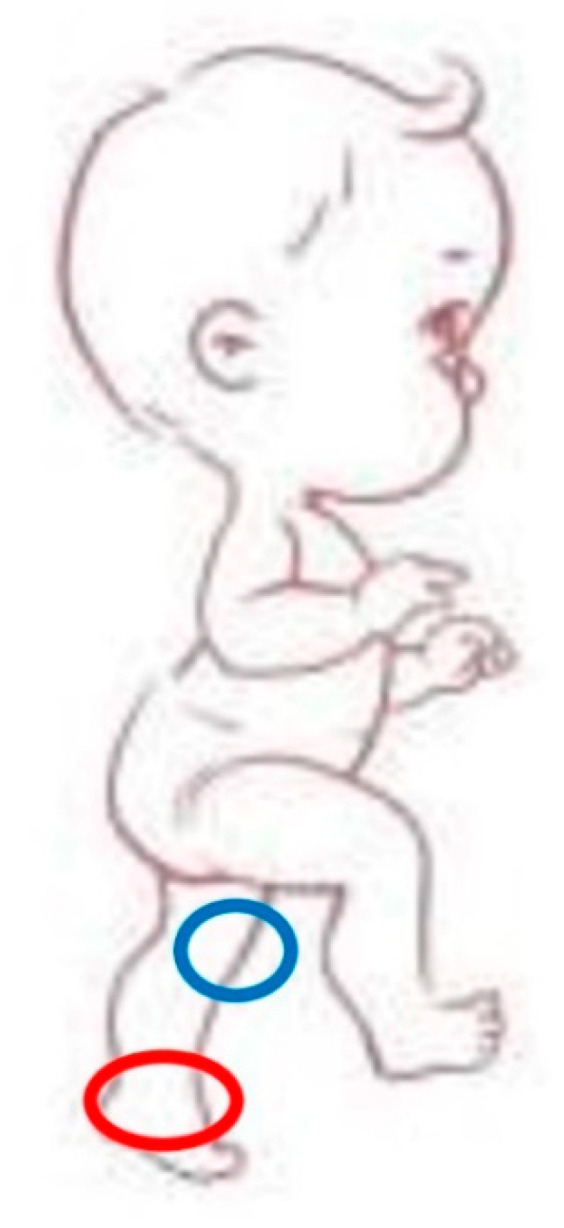
The figure shows how during walking the dysfunctions are mainly borne by the ankles, which contract (red circle), and the knees which hyperextend (blue circle).

The main stages of infant motion development [7,11], can be summarized as follows:

- Tummy time: when the infant is placed on his stomach while awake with someone watching.
- Rolling: the first transitional motion skill that allows the infant to begin exploring his world and use together both sides of the body so that he can learn how to use arms and legs together (a prelude to creeping and crawling).
- Creeping: this movement allows the infant to attain greater control of his body than rolling and offers him a greater possibility of interacting with the surrounding environment. Among the different ways of moving horizontally, this is the most tiring, even though some children become very skilled.
- Pivoting (or clock shifting): this movement consists of the ability of the infant, in the prone position, to rotate in both directions, by pivoting with a part of the body, usually an arm.
- Shuffling: this movement, also known as ‘movement on the bottom’, is not a particularly frequent step of development but, if present, tends to be long-lasting, because it is advantageous compared to the others. Gaze is already in an optimal position, hands are free to grasp, explore, and transfer the surrounding objects; there is no fear of instability and falls because the infant is already mastering the sitting position.
- Crawling: it is not a mandatory stage for access to walking, yet it is usually very frequent and of considerable importance for motion coordination. The infant who starts to crawl moves forward with hands and knees as support, the lower limbs remaining flexed. This gait can be: (a) ipsilateral symmetrical when the upper and lower limbs of the same side move at the same time; (b) cross asymmetrical when the upper and lower limbs of the different sides move together, thus preparing the future cross pattern of walking. During this step, knees and elbows are the most stressed joints (in red and blue in Figure 1).

- Standing position: the infant moves from lying to sitting without assistance, stands up with one foot forward, and begins to walk with two hands held, usually around the first year of age.
- Walking: the infant crawls up the stairs, comes off the floor without support, and begins to walk well on his own. He also squats and stands up without holding on to any support. During the walk, the dysfunctions are mainly borne by the ankles, which contract (red circle), and the knees which hyperextend (blue circle), as shown in Figure 2.

In the infant with hemophilia, different joint stresses favor the onset of bleeding. Table 1 lists the site of the first bleed based on the type of movement and developmental stage of the child, the sites of subsequent bleeds due to the involvement of the corresponding kinematic chain as well as suggestions on which signals parents must be able to grasp to avoid or control bleeding events.

## 4. How the Clinician Should Act to Prevent Joint Damage

The joint damage typical of hemophilia ultimately causes irreversible deformity and disability [10]. To avoid this, it is necessary to prevent bleeding as much as possible starting from infancy. The early use of prophylactic therapy with coagulation factor concentrates or innovative drugs such as emicizumab has dramatically reduced the number of muscle and joint bleeds [12,13] but without fully eliminating them. Aiming for zero bleeds is the goal of modern hemophilia therapy, but zero bleeding may not be enough to prevent joint damage. Tackling subclinical bleeding should be the aim of a truly effective therapy. Clinicians, nurses, and physiotherapists must therefore better inform parents on how to observe their child, to grasp any slightest sign of an underlying problem (pain, micro-bleeding, synovitis, etc.) and thus immediately intervene by interrupting a vicious circle of events ultimately causing joint damage. We suspect that subclinical joint bleeding in the infant can also be caused by abrupt movements during dressing or diaper change. Particular attention should thus be paid to the position that an infant enters the space with, to their preference for one side of his body rather than the other, and to the more harmonious movements from one side because infants and children may not report pain by crying or talking but rather by avoiding the painful position or refusing to walk or perform certain activities if these generate pain. The collection of this information in a diary will help physiotherapists and hematologists recognize very early joint involvement and correct these dysfunctions, helping the child to perform correct motion gestures, encourage motion on both sides, and stimulate balance using sounds, games, and objects that intrigue the child. The parents must be able to grasp any small abnormal sign (inexplicable cries, incoherent movements, avoiding walking or using a limb, support on a single side, etc.) to immediately report it to the people who are treating the child, to be able to immediately intervene and avoid the occurrence of damage.

In addition, the evaluation of joint status by ultrasound and physical examination since the first referral of the patient and before starting the primary prophylaxis will lead to strict joint health monitoring and collection of data for future studies. Shared decision making on the opportunity of an earlier introduction of replacement or non-replacement therapies and the patient or parents’ preference should be considered in each Hemophilia Care Center; finally, we suggest the early involvement of physiotherapists in the assistance of infants, concretely teaching the parents for avoiding stress in each motor activity of the infant.

## 5. Limitations and Future Directions

What is written in this manuscript is the result of the authors’ experience, gained over many years of activity in treating and managing hemophilia patients. It is therefore an “expert opinion” based on the observation of these subjects throughout their lives, with particular attention to childhood, where some musculoskeletal dysfunctions begin to manifest themselves and where it is also easier to intervene to prevent the development of disabilities and deformities.

There are currently no studies available in this regard, but it is hoped that these observations of ours can be the starting point to design some to better understand the risk factors involved in joint stress in childhood, and the role of prophylaxis and of early behavioral interventions in the prevention of joint bleeding, altered mobility, and finally hemophilic arthropathy in adults.

## 6. Conclusions

Careful observation since the birth of the motion gestures of the child with hemophilia and the rapid recovery in the case of dysfunction are together with early prophylaxis implementation the appropriate synergistic strategies in order to avoid even the smallest joint damage in hemophilia.

## Figures and Tables

**Table 1:** The site of the first bleed based on the type of movement and developmental stage, the sites of subsequent bleeds, and the suggestions for parents.

Motion Development Steps	Age (Months)	Sites of First Bleeding	Corresponding Kinematic Chain and Possible Sites of Subsequent Bleeds	Suggestions
Tummy time	0–3	Knees, ankles, elbows	Shoulders, hips, iliopsoas	Pay attention to how you hold the child when dressing, bathing, or changing their diaper
Rolling	3–6	Elbows, knees, iliopsoas	Shoulders, hips	Alternating left and right rolling
Creeping	6–9	Knees, elbows	Wrists, shoulders	Encourage the child to use both sides of the body. Observe if he uses them equally
Pivoting	6–9	Elbows	Shoulders, wrists	Encourage the child to use both sides of the body. Observe if he rotates on both sites.
Shuffling	6–9	Knees	Hips, iliopsoas	Pay attention to one-sided shuffling
Crawling	9–12	Knees, elbows	Shoulders	Pay attention to one-sided crawling
Standing with help	9–12	Knees, ankles	Feet (support area), iliopsoas, lumbar spine, hips	Stimulate proprioception of the feet
Walking	12–14	Ankles, knees	Feet, iliopsoas, backbone, hips	Stimulate balance
